# Hydrodynamic and geometric effects in the sedimentation of model run-and-tumble microswimmers

**DOI:** 10.1039/d1sm01594j

**Published:** 2022-02-16

**Authors:** Andrea Scagliarini, Ignacio Pagonabarraga

**Affiliations:** a IAC-CNR, Istituto per le Applicazioni del Calcolo “Mauro Picone” Via dei Taurini 19 00185 Rome Italy andrea.scagliarini@cnr.it; b INFN, Sezione Roma “Tor Vergata” Via della Ricerca Scientifica 1 00133 Rome Italy; c CECAM, Centre Européen de Calcul Atomique et Moléculaire, Ecole Polytechnique Fédérale de Lausanne Batochimie, Avenue Forel 2 1015 Lausanne Switzerland; d Departament de Física de la Matèria Condensada, Universitat de Barcelona Carrer de Martí i Franquès 1 08028 Barcelona Spain; e Universitat de Barcelona, Institute of Complex Systems (UBICS), Universitat de Barcelona 08028 Barcelona Spain

## Abstract

The sedimentation process in an active suspension is the result of the competition between gravity and the autonomous motion of particles. We carry out simulations of run-and-tumble squirmers that move in a fluid medium, focusing on the dependence of the non-equilibrium steady state on the swimming properties. We find that for large enough activity, the density profiles are no longer simple exponentials; we recover the numerical results through the introduction of a local effective temperature, suggesting that the breakdown of the Perrin-like exponential form is a collective effect due to fluid-mediated dynamic correlations among particles. We show that analogous concepts can also fit the case of active non-motile particles, for which we report the first study of this kind. Moreover, we provide evidence of scenarios where the solvent hydrodynamics induces non-local effects which require the full three-dimensional dynamics to be taken into account in order to understand sedimentation in active suspensions. Finally, analyzing the statistics of the orientations of microswimmers, the emergence of a height-dependent polar order in the system is discussed.

## Abstract 2

The shape of the steady state of sedimentation profiles of an active suspension depend crucially on the competition between gravity and activity as well as on collective effects determined by solvent hydrodynamics and system geometry.

## Graphical abstract

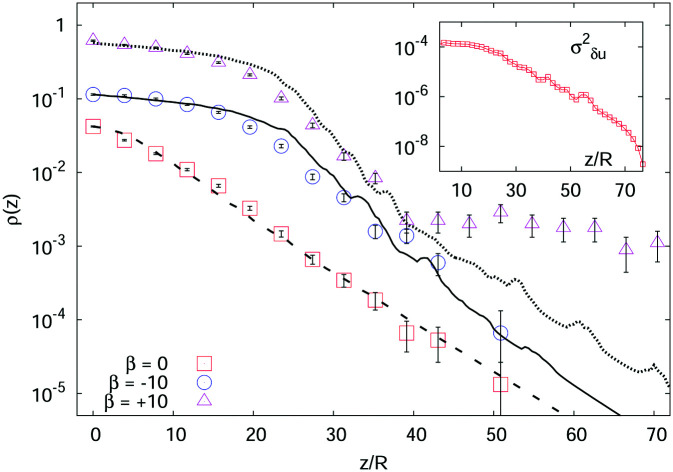

## 1 Introduction

A number of microorganisms (bacteria, algae, *etc*.…) have the ability to swim in a liquid environment through the generation of autonomous motion at the expense of their metabolism, thus being intrinsically out-of-equilibrium. As such, these systems lead to new challenges such as the understanding of how collective phenomena and self-organization emerge from the relevant features of the propulsion mechanism.^1–4^ In this perspective a suspension of active particles is qualitatively different from a suspension of passive ones. Maybe the simplest, yet not trivial, example of this is the case of a constant external forcing on the suspension, such as gravity in the sedimentation process. In fact, when thermal fluctuations are negligible (as in the case of particles above the micron size), while passive particles would inevitably precipitate, active suspensions maintain a finite sedimentation length that grows with the self-propulsion speed. This result was predicted theoretically for *dry* suspensions (*i.e.* where the solvent hydrodynamics is neglected) of non-interacting run-and-tumble particles^5,6^ and, then, confirmed in numerical simulations with point-like dipoles^7^ and experimentally in suspensions of active colloids.^8^ Suspensions of self-propelled particles under gravity have been also reported to display a complex orientational dynamics, with the development of an associated polar order^9,10^ or even, in the case of bottom-heavy particles, to the inversion of the sedimentation profiles.^11^ In this paper we present a computational study of sedimentation in active suspensions, where hydrodynamics is fully resolved near and far from the particle surface. We provide evidence that hydrodynamic correlations induce important deviations from the phenomenology for dry suspensions in the steady state of both self-propelled swimmers and “shakers”, namely active particles that stir the fluid around them without achieving a directed motion, for which, to the best of our knowledge, this study represents the first of this kind. The sedimentation profiles observed when activity is intense are captured through a simple extension of a drift-diffusion model with height dependent effective temperature. We show that pullers develop a distal region of constant density (a supernatant) whose emergence depends on both the activity/gravity ratio and the confining geometry (*i.e.* the cell aspect-ratio). We also address the statistics of the microswimmer orientation, finding that, in the regime of small tumbling frequency, the suspension develops a polar order whose characteristics are strongly dependent on the type of swimmer.

## 2 Numerical method and simulation details

The velocity field of the solvent (of dynamic viscosity *η*) is evolved by means of a lattice Boltzmann (LB) method^12^ with nineteen lattice speeds in three dimensions (D3Q19).^13^ Swimmers are modelled as solid spherical objects of radius *R*. The correct momentum exchange and mass conservation through the set of boundary links (between grid points in and out the sphere) representing the particles is implemented according to the bounce-back-on-links scheme.^14–16^ In order to mimic the surface deformations inducing the microswimmer motion, we adopt a simplified version of the squirmer model,^17,18^ whereby only the tangential polar component of the axisymmetric velocity prescribed at the particle surface is non-zero, **u**_s_ = *u*_s_(*θ*)*θ̂*. Furthermore, just the first two terms in the series expansion of *u*_s_(*θ*) are retained:^19,20^

**Fig. 1:**
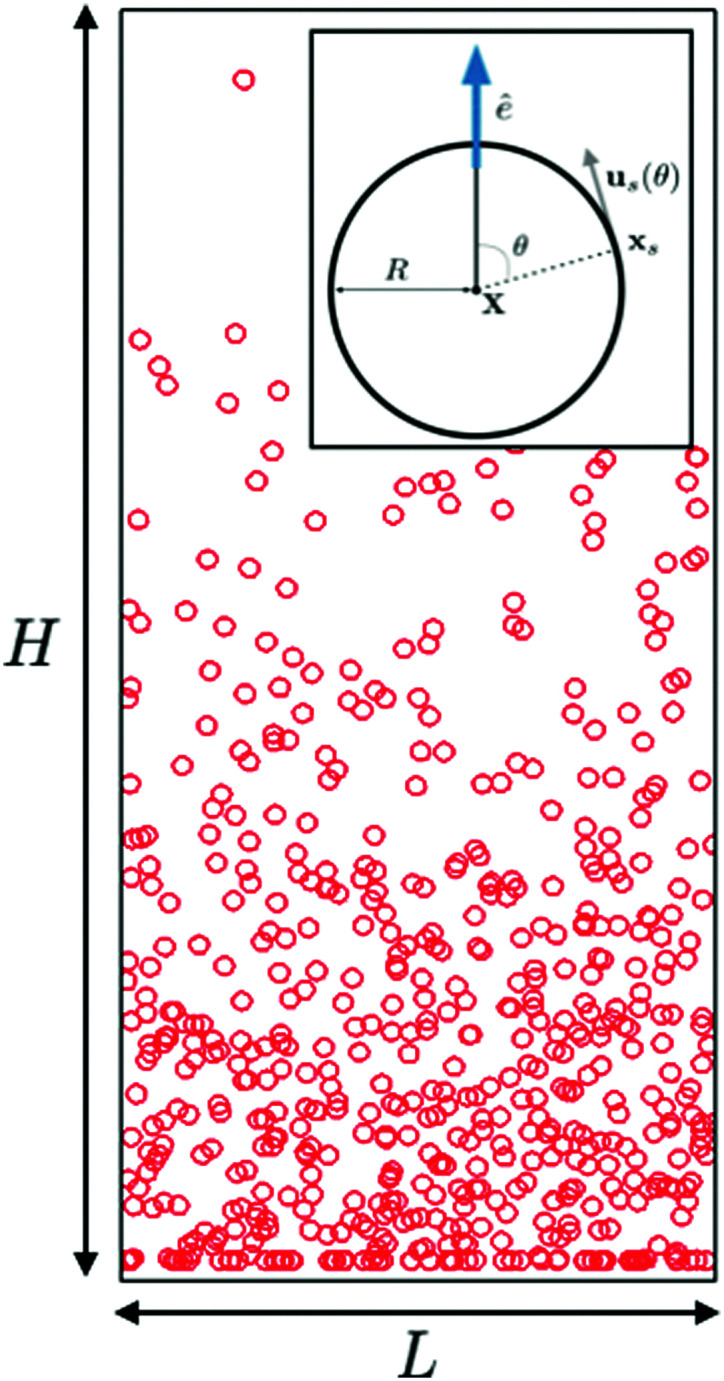
Snapshot of a simulation, seen on the (*x*, *z*) plane, in the statistically steady state; *L* and *H* indicate the sizes of the box, *L* × *L* × *H*. Inset: Sketch of a squirmer particle of radius *R*. *X* is the position of the centre of mass, *x*_s_ is the position on the surface, *ê* is the characteristic orientation unit vector, defining the appropriate swimming direction, and the polar angle reads 
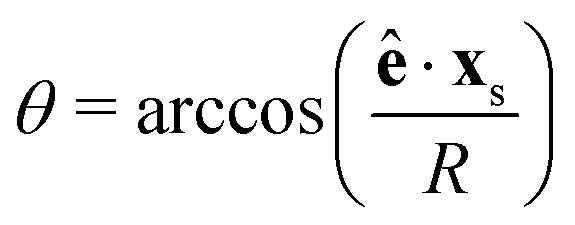
.

1 *u*_s_(*θ*) = (*B*_1_ + *B*_2_ cos(*θ*))sin(*θ*),

where *θ* = arccos(**ê**·**r̂**_s_) is the angle formed by the squirmer orientation unit vector, **ê**, and the position on the surface, **r̂**_s_ = **x**_s_/*R*, relative to the particle centre of mass position (see Fig. 1 for a schematic representation of the model microswimmer just described). It should be pointed out that the prescription (1) cannot cope with unsteady flows, like those occurring when flagellar beating is involved, in short times, but it is effective on scales much longer than a typical flagellar or ciliary cycle.^21^ The parameter *B*_1_ > 0 in eqn (1) is related to the propulsion speed, which is 
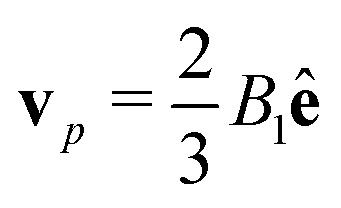
, whereas the second parameter, *B*_2_, determines the strength of the stresslet, 𝒮 ∝*ηR*^2^*B*_2_, generated by the swimmer in the surrounding fluid (and, hence, it is related to the amplitude of the injected vorticity).^19^ The ratio 
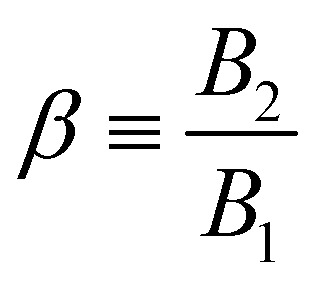
, such that *β* ∈ (−∞, + ∞), quantifies the relative intensity of apolar stresses and polar self-propulsion and classifies swimmers in “pushers”, *β* < 0 (including bacteria like, *e.g., E. coli*), “pullers”, *β* > 0 (such as the algae *Chlamydomonas*), and “potential” swimmers, *β* = 0 (*i.e.* swimmers that simply self-propel without generating vorticity, like the alga *V. carteri* or certain artificial swimmers).^17,19,22–24^ Every *τ* time step the particles randomise their orientation **ê** with uniform probability over the interval [0, π], thus accounting for the characteristic run-and-tumble mechanism, which can be seen as a source of diffusion for particles that, we recall here, are insensitive to thermal fluctuations.^25,26^ Different probability distributions of tumbling angles can, in principle, characterise actual microswimmers. For *E. coli*, for instance, the distribution is peaked around ∼65° and is rather skewed towards smaller values.^25^ It is known, though, that, when looked at over time scales *t* ≫ *τ*, the run-and-tumble motion leads to a diffusive dynamics, irrespective of the specific statistical properties of the tumbling events, but for a dependence of the diffusion coefficient on the mean angle.^25,27–29^

Our model, featuring finite size resolved particles, equipped with the squirming motion, is then able to capture hydrodynamic effects in the sedimentation of active suspensions, both in their far and near field manifestations, although when particles are close to contact or swimming takes place near the walls, the dynamics on short time scales might be not accurately described for flagellated microorganisms.

We simulate suspensions, of volume fraction *ϕ* = 0.07, in three-dimensional boxes of size *L* × *L* × *H*, with height *H* ≈ 80*R* and variable aspect-ratio *Γ* = *L*/*H* (see Fig. 1 for a graphical sketch). The height value is chosen to be large enough to exceed the maximum theoretically expected sedimentation length (over the explored range of parameters and for cases where such theoretical control is available), so as to guarantee that the upper bound will not affect the results. Two solid walls (with no-slip boundary conditions for the fluid velocity) confine the system in the *z*-direction, while periodic boundary conditions along the *x*, *y* directions hold. The number of particles, with radius *R* = 2.3 (in lattice-spacing units), range between ∼500 and ∼3 × 10^4^). We introduce a reference velocity, *v*_*g*_ = *μF*_*g*_ (where *F*_*g*_ is the gravity force magnitude and *μ* = 1/(6*πηR*) is the particle mobility), *i.e.* the sedimentation velocity of a passive particle, and a reference time, *t*_*c*_ = *R*/*v*_*p*_ (where **v**_*p*_ = |*v*_*p*_|), that is basically the time an isolated particle takes to displace its own radius. In terms of *v*_*g*_ and *t*_*c*_, the following dimensionless parameters can be defined, namely2
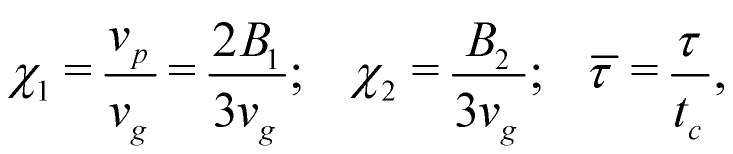
which, together with *β*, govern the squirmers’ motion. In order to investigate how the swimming characteristics and the system geometry affect the sedimentation profiles, we performed several runs exploring the parameter space spanned by (*χ*_1_, *χ*_2_, *β*, *Γ*). Unless differently specified, we fix *τ̄* ≈ 4.3, corresponding to a run time much longer than the typical time the flow field takes to relax and adapt to the new orientation; the latter, given the low Reynolds number dynamics, can be taken as *t*_*ν*_ ∼ *R*^2^/*ν*, the viscous time of diffusion around the particle (*ν* being the solvent kinematic viscosity), so that *τ*/*t*_*ν*_ ≈ 30.

## 3 Sedimentation profiles

We start each run with the active particles homogeneously distributed in space, with random orientations. To check that a (non-equilibrium) statistically steady state is reached, we follow the time evolution of the average height 
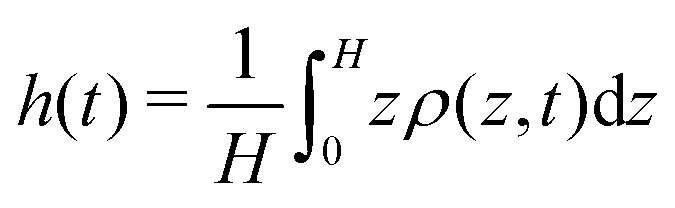
, where *ρ*(*z*, *t*) is the (unsteady) normalized particle density (*i.e. ρ*(*z*, *t*)d*z* is the probability of finding a particle centred between *z* and *z* + d*z* at the time *t*). We consider as the steady state the time interval during which *h*(*t*) fluctuates by less than ∼5%. All data shown hereafter are meant to be averaged over such time interval. Our aim is to study the impact that activity, in terms of *χ*_1_ and *β*, has on the squirmer sedimentation, and to characterize the emerging dynamical regimes, checking whether and how hydrodynamic effects come into play. According to the theory,^5,6^ as *χ*_1_ → 1, all particles concentrate at the bottom wall. Instead, when *χ*_1_ ≫ 1 (*i.e.*, in the self-propulsion dominated regime) the steady state sedimentation profile should display an exponential form *ρ*(*z*) ∼ *e*^−*z*/*λ*^, with a sedimentation length depending on the single particle velocity (and, hence, on *χ*_1_) as3
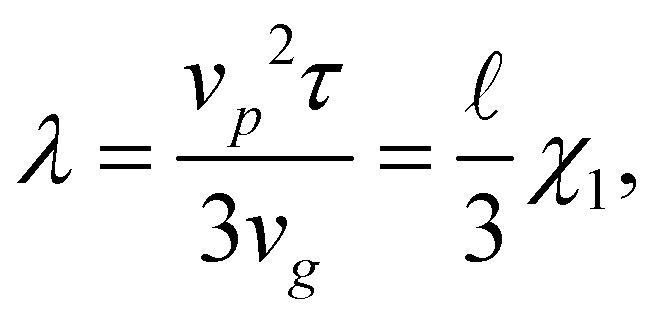
where *ℓ* = *v*_*p*_*τ* ≈ 4.3*R* ≈ 0.06*H* is the microswimmer's run length. This result has been found to be in agreement with experimental observations^8^ and numerical simulations.^7^ The exponential profile also characterizes equilibrium systems, as in the classical Perrin's experiment for (thermal) colloids;^30^ the sedimentation length is determined by the particle diffusivity, *D*, and the gravity force as *λ*^(eq)^ = *D*/(*μF*_*g*_) and depends, therefore, through the Stokes–Einstein relation *D* = *μk*_B_*T*, on the system temperature *T*, namely *λ*^(eq)^ = *k*_B_*T*/*F*_*g*_. The formal analogy with the passive (equilibrium) case suggests, then, to introduce an effective temperature as follows:4
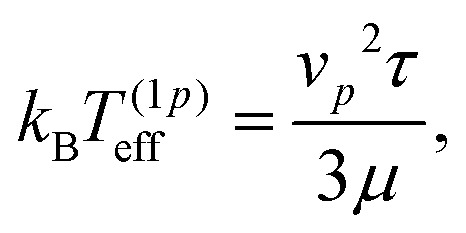
such that the sedimentation length reads *λ* = *k*_B_*T*^(1*p*)^_eff_/*F*_*g*_.

**Fig. 2:**
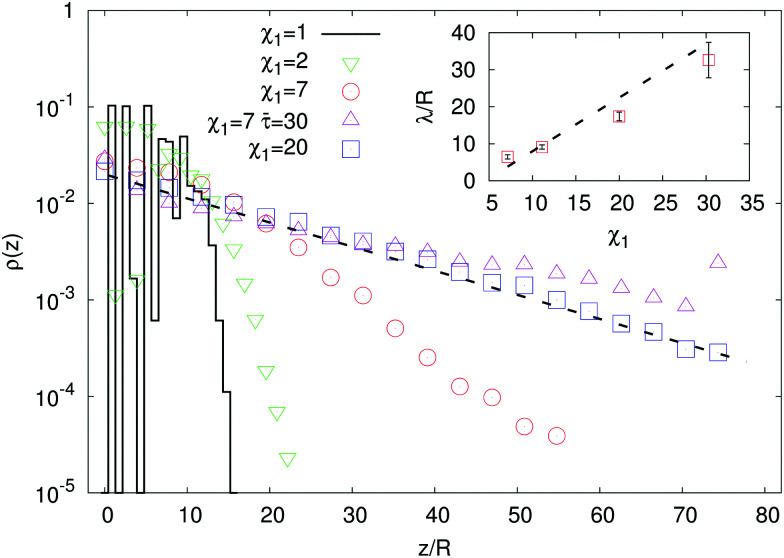
Main panel: Density profiles in microswimmer suspensions for various values of the propulsion/gravity ratio *χ*_1_, at *β* = 0 and *Γ* = 0.35. For *χ*_1_ close to one, the particles accumulate at the bottom wall, showing a crystal order (as the regularly spaced peaks in *ρ* suggest). For large *χ*_1_ the expected exponential profile is recovered. Inset: Dependence of the sedimentation length *λ* (computed out of exponential fits of the density profiles) (□) on the propulsion/gravity ratio *χ*_1_. The dashed line depicts the theoretical expectation *λ*/*χ*_1_ = *ℓ*/3 ≈ 1.45*R*, eqn (3), valid for *χ*_1_ ≫ 1.

In Fig. 2 we plot the time-averaged steady state density profiles of potential swimmers (*β* = 0) for *χ*_1_ ∈ [1, 20]. This range of values is compatible with those expected for typical bacteria, such as *E. coli* or *B. subtilis* whose swimming speeds are *v*_*p*_ ∼ 15–30 μm s^−1^,^25,31,32^ in terrestrial gravity (*v*_*g*_ ∼ 1–2.5 μm s^−1^), for which it would be *χ*_1_ ∼ 6–30.^6^

For values close to one, as expected, microswimmers uniformly fall down under the action of gravity; however, due to the finite size of particles, the sedimentation length remains finite. The particles in the sediment tend to organize themselves in layers with a crystal-like order, noticeable from the peaks in the density profile, close to the bottom wall, displaced from each other by about one diameter (2*R*), as found also in a previous computational study.^10^ At increasing *χ*_1_, swimmers occupy an increasingly larger volume of liquid and, correspondingly, *ρ*(*z*) shows, over the whole box length, the predicted exponential profile^6^ with a sedimentation length growing linearly with *χ*_1_ (see inset of Fig. 2).

**Fig. 3:**
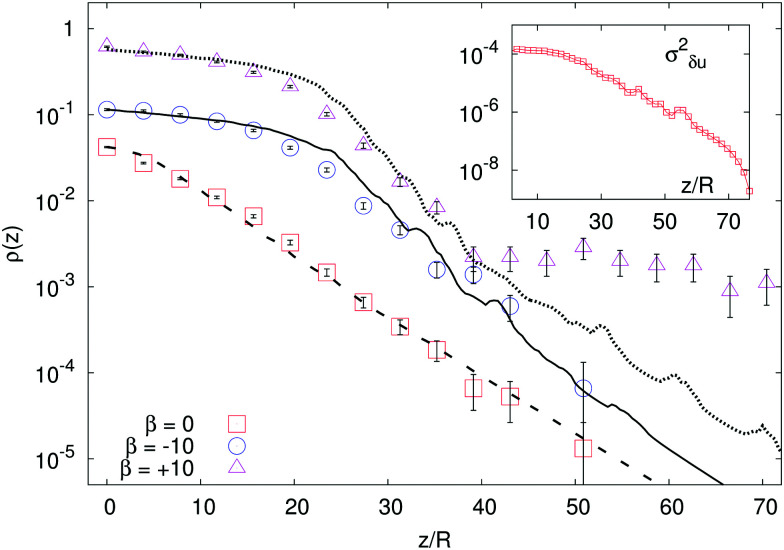
Main panel: Density profiles in microswimmer suspensions with *χ*_1_ = 10, *β* = 0, ±10 and *Γ* = 0.35 (data are vertically shifted for clarity). The lines represent the predictions coming from the numerical integration of eqn (7) with *λ* = 15 and *α*_1_ = 1 (see the text for the discussion of the model parameters) for *β* = 0 (dashed line), *β* = −10 (solid line) and *β*= + 10 (dotted line). Inset: Fluid velocity fluctuations 
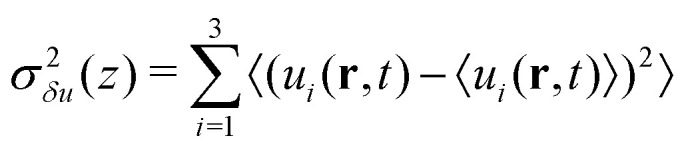
 as a function of the system height for the case *β* = − 10.

If we increase |*β*| (thus intensifying the activity) to large enough values, for a fixed *χ*_1_, the deviation from the exponential profile can be important, as one can see from Fig. 3, where we plot the particle density *ρ*(*z*) for three cases with same *χ*_1_ = 10 and *β* = 0, ±10. For the sake of comparison of the chosen values of *β* with those expected for actual microswimmers, consider that, *e.g.*, *E. coli* swims at a speed *v*_*p*_ ∼ 20 μm s^−1^,^25^ exerting a force dipole of amplitude *f* ∼ 0.4 pN and length *δ* ∼ 2 μm;^31^ therefore, 
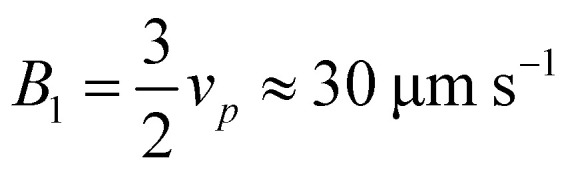
 and |*B*_2_| ∼ 𝒮/(*ηδ*^2^) ∼ *f*/(*ηδ*) ≈ 200 μm s^−1^, where 𝒮 ∼ *fδ* is the stresslet, give |*β*| = |*B*_2_|/*B*_1_≈7.

In the pushers/pullers case (*β* ≠ 0), dynamic correlations are so intense that recovering a Perrin-like form just with the introduction of a global effective diffusion coefficient as coming from single particle dynamics is no longer possible.^8^ The larger the |*β*|, the stronger is the departure of the sedimentation profile from being exponential; indeed we found that deviations start to be relevant from |*β*| ≈ 5.^7^

## 4 Extended diffusive model

### 4.1 Local effective temperature

Due to hydrodynamic correlations the dynamics of an active particle in the suspension is affected by the presence of the others through the generation of motion within the liquid, which acts as a bath at an effective temperature (measuring the fluid agitation). We can understand these effects extending a diffusive model proposed to describe sedimentation in active colloidal suspensions,^8^ based on the Smoluchowski equation ∂_*t*_*ρ* = −∇·**J**, determined by the flux **J** = −*D̃*∇*ρ* + *μ̃***F**_*g*_*ρ*. The ratio of the *local* diffusion coefficient, *D̃*, and particle mobility, *μ̃*, by virtue of a generalized Stokes–Einstein relation, represents the effective temperature field. Assuming that in the steady state the density will depend only on *z*, the zero flux boundary conditions at the walls gives5
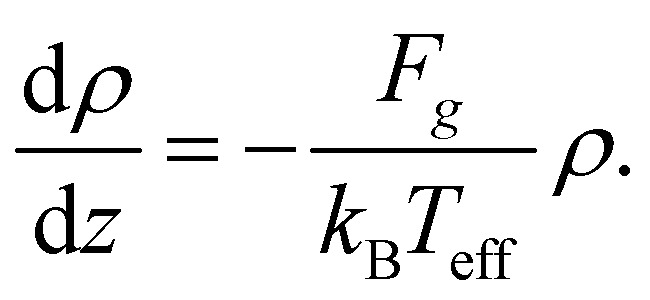


We propose an effective temperature of the form *T*_eff_ =*T*^(1*p*)^_eff_ + *T*^(coll)^_eff_, consisting of two terms: the single-particle effective temperature, eqn (4), accounting for the self-propulsion, plus a contribution proportional to the fluid velocity fluctuations, *T*^(coll)^_eff_, capturing the collective effects due to hydrodynamic interactions. However, since in the steady state microswimmers are distributed inhomogeneously over the volume (with a density increasing from top to bottom), the fluid velocity fluctuations 
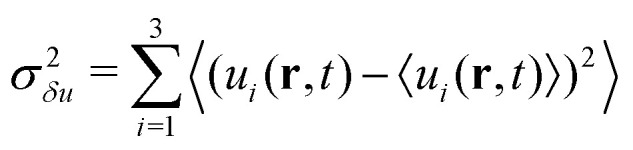
 (where 
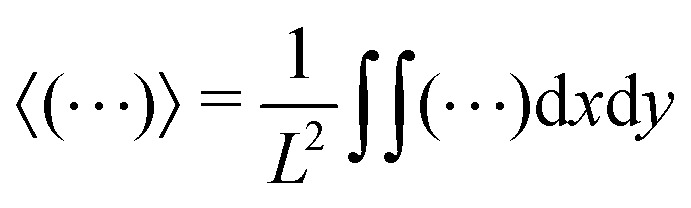
) are also expected to vary with *z* (as indeed it can be seen in the inset of Fig. 3). This entails a height dependent effective temperature *T*_eff_(*z*) = *T*^(1*p*)^_eff_ + *T*^(coll)^_eff_(*z*), leading, upon insertion in (5), to an equation for the sedimentation density which can be recast in the following form:6
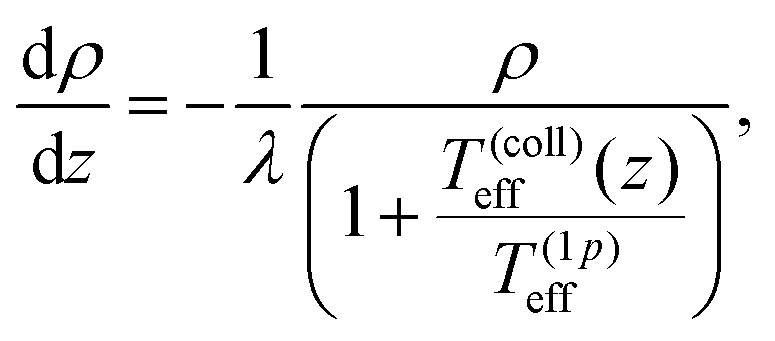
where *λ* = (*k*_B_*T*^(1*p*)^_eff_)/*F*_*g*_ is the sedimentation length discussed in the previous section. We assume, then, *T*^(coll)^_eff_(*z*) ∝*σ*_*δu*_^2^(*z*) to hold, so that we can finally write7
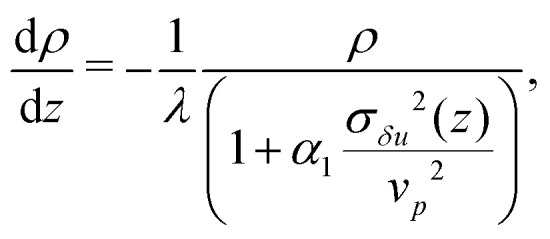
with *α*_1_ a free parameter representing the proportionality constant between *T*^(coll)^_eff_ and *σ*_*δu*_^2^. Eqn (7) is integrated numerically, with *σ*_*δu*_^2^ taken from the simulations. Comparing the result with the measured density profiles (see Fig. 3), we find that the proposal of gauging the global effective temperature to a height dependence works well for *β* = 0 and *β* < 0. The phenomenology of pullers (*β* > 10) appears, however, to be more complicated: in fact, while the density profile can be recovered where the concentration is higher, the presence of a region of constant density, denoting the formation of a supernatant floating over the sedimentation layer, eludes the generalized diffusive model.

### 4.2 The case of shakers

**Fig. 4:**
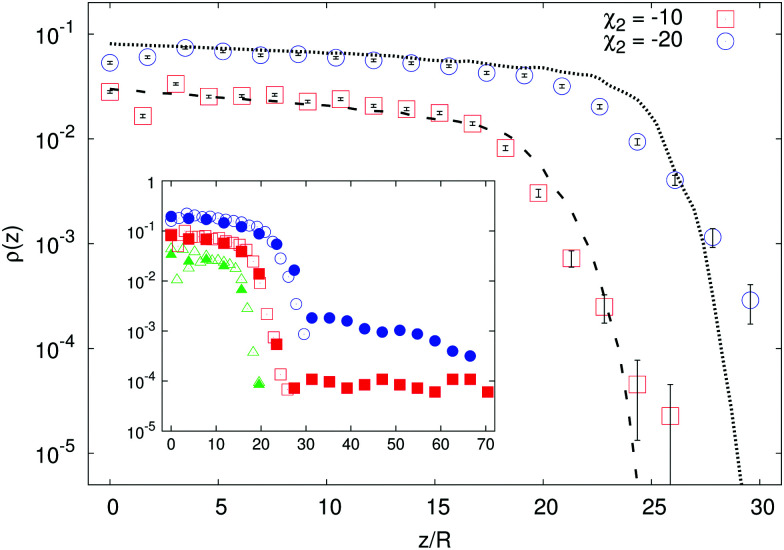
Main panel: Density profiles of shakers with two different *χ*_2_ < 0 and *Γ* = 0.35 (here and in the inset data are vertically shifted for clarity). The larger the |*χ*_2_| the longer the density tail (*i.e.* the wider is the region occupied by particles). The lines are the theoretical predictions coming from the numerical integration of eqn (8), where the function *σ*_*δu*_^2^(*z*) is taken from the simulations, with *α*_2_ = 4.4. Inset: Density profiles of shakers with |*χ*_2_| = 5 (green triangles), |*χ*_2_| = 10 (red squares) and |*χ*_2_| = 20 (blue circles). Data for both pullers (*χ*_2_ > 0, full symbols) and pushers (*χ*_2_ < 0, empty symbols) are reported; notice the formation of the supernatant in the puller case for large enough *χ*_2_.

Another striking instance of how crucial the role played by hydrodynamics can be is provided by the regime where |*β*| → ∞, *i.e. B*_1_ goes to zero while *B*_2_ stays finite. This regime corresponds to active suspensions where particles do not self-propel but generate motion in the fluid and are relevant for microswimmers known as shakers,^1,33^ like, *e.g.*, melanocytes.^34^ Since both their propelling velocity and the effect of thermal fluctuations are negligible, such a suspension would undergo a gravitational collapse, if one could completely neglect the presence of the solvent. However, as shown in Fig. 4, the steady state density profiles develop a sedimentation layer, whose width increases with *χ*_2_ (defined in (2)). The observed width cannot be interpreted simply as a result of the close packing of the particles, which would imply, in fact, a value of around 8*R*, much smaller than the measured one. We try to recover the sedimentation profiles of shakers following the same ideas of the previous section. We must integrate numerically eqn (5), with a vanishing one-particle contribution to the effective temperature, *T*^(1*p*)^_eff_ = 0, since for shakers *B*_1_ = 0 ⇔ *v*_*p*_ = 0, such that *T*_eff_(*z*) = *T*^(coll)^_eff_(*z*) = *α*_2_(*σ*_*δu*_^2^(*z*)/*v*_*B*2_^2^) (here we indicate the phenomenological parameter as *α*_2_ in order to distinguish it from that of self-propellers). The reference speed *v*_*B*2_ = |*B*_2_|/3 is the magnitude of the velocity field generated by a shaker, averaged over its surface, *i.e.*
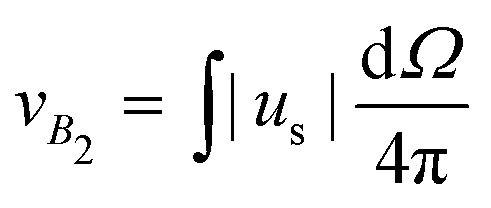
, where *u*_s_ is given by eqn (1). The stationary Smoluchowski equation, then, reads8
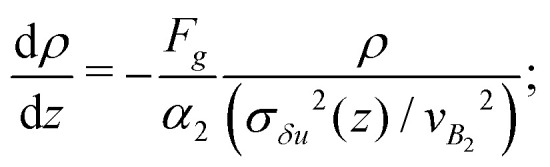
the results of the numerical integration of eqn (8) for shakers with negative *χ*_2_ with two different values of gravity are reported in Fig. 4, showing, again, good agreement.

**Fig. 5:**
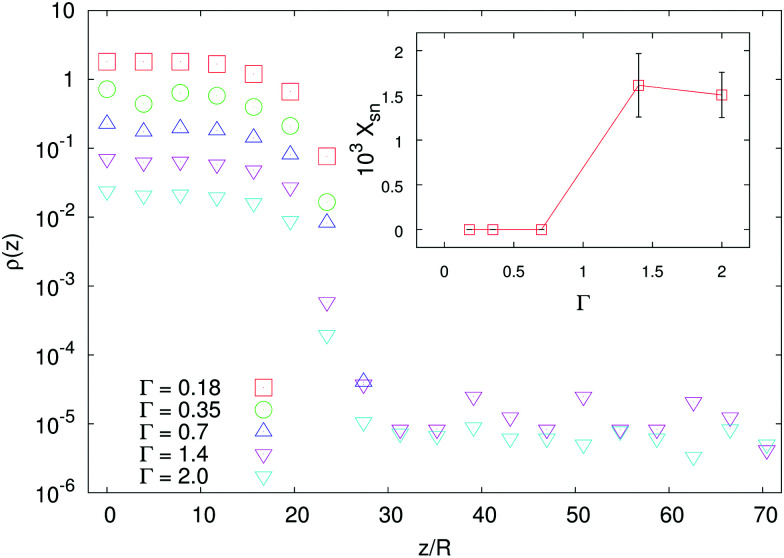
Main panel: Density profiles for shakers with *χ*_2_ = 8.3 for various aspect ratios *Γ* = *L*/*H* (data are vertically shifted for clarity). Inset: Fraction of particles in the supernatant region, computed as 
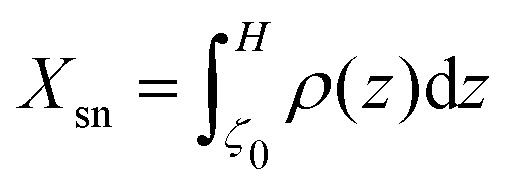
 (*ζ*_0_ being the minimum height such that *ρ*(*z*) = 0, for some *Γ* and for *z* > *ζ*_0_), as a function of the aspect ratio of the cell: notice that for *Γ* < 1, *X*_sn_ = 0, *i.e.* no supernatant develops.

Analogously to the case of pullers, shakers with *χ*_2_ > 0 develop (for *χ*_2_ large enough) a distal region of constant density in the sedimentation profile (see the inset of Fig. 4). The emergence of such supernatant is due to the sediment which acts as a pump and generates motion in higher layers of fluid. It is, then, a genuinely three-dimensional and non-local effect, two features which make also our formalism based on a height dependent effective temperature fail. To support this picture, we show that, for a fixed value of *χ*_2_, the supernatant disappears when decreasing the aspect-ratio *Γ* of the cell below unity (see Fig. 5).

This is, indeed, a manifestation of three-dimensionality: using an analogy with a Rayleigh–Bénard system,^35^ we argue that the geometry favors (or does not) the development of a large scale flow which can (or cannot) sustain the supernatant. In fact, the difference in the fluid flow pattern generated by a single particle, either a pusher or a puller, is not strong enough to sustain the different macroscopic patterns observed if the swimmers are randomly oriented (as a matter of fact, no supernatant is observed for pushers, or shakers with *χ*_2_ < 0). Hence, a collective organization of the swimmers is required to produce the observed macroscopic flows. We will next address the emergence of orientational order in the sedimentation profiles of microswimmers.

## 5 Orientational statistics

**Fig. 6:**
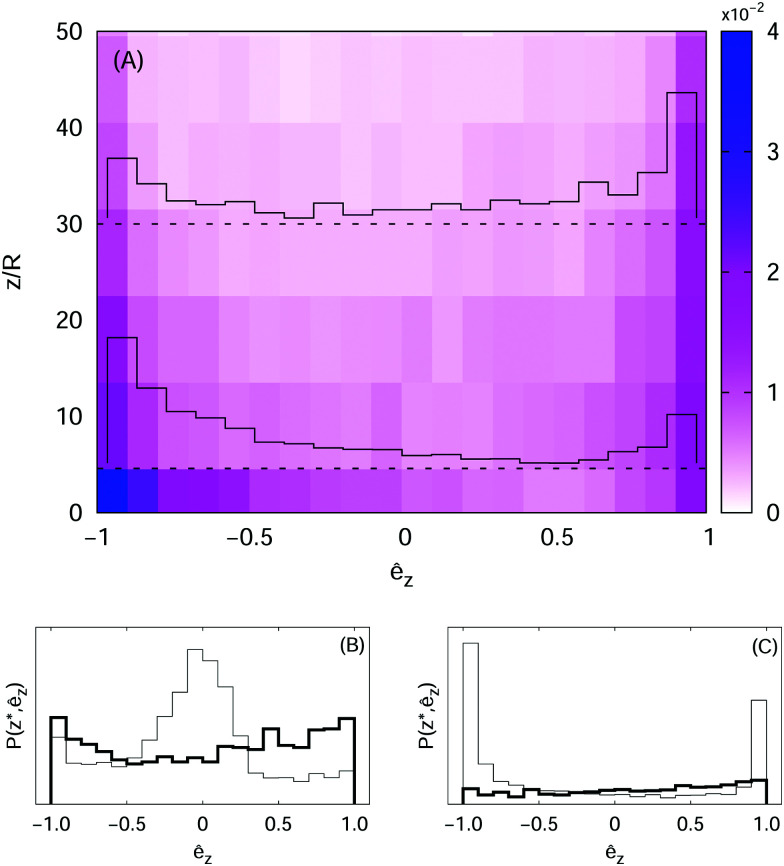
Panel (A): Joint probability distribution function, *P*(*z*, ê_*z*_), of elevation, *z*, and vertical component of the orientation unit vector, ê_*z*_, for microswimmers with *β* = 0; the histograms depicted as continuous lines highlight the orientation PDF, *P*(z*, ê_*z*_)/*ρ*(z*), at two different elevations: z* = 2*R* and z* = 30*R* (indicated with dashed lines). Panel (B): Orientation PDFs for pushers*, β* = −10, at z* = 2*R* (thin line) and z* = 30*R* (thick line). Panel (C): Same as panel (B) but for pullers, *β* = +10.

The emergence and the dynamical relevance of anisotropic ordering in active fluid systems has been widely recognized in the literature.^1,9,36^ We study the orientational statistics measuring the joint probability distribution function (PDF) of the particle elevation and vertical component of the squirmer characteristic vector, *P*(*z*, ê_*z*_). For squirmers with *χ*_1_ = 10, *β* = 0 and run time *τ̄* ≈ 4.3 we find a bimodal distribution symmetrically peaked at ê_*z*_ = ±1 (with a slight imbalance towards ê_*z*_ = −1), for any *z*, as expected under the assumption of a factorized joint PDF, *P*(*z*, ê_*z*_) ∼ *ρ*(*z*)*Φ*(ê_*z*_).^26^ However, it was shown theoretically, in the context of active Brownian particles, that such factorization could only be possible for the vanishing Péclet number;^9^ otherwise, when Pe ∼ *O*(1), the suspension develops a polar order which is non-trivially correlated with the height. We recall, here, that for athermal, run-and-tumble particles an effective diffusivity can be defined, proportional to the tumbling rate, *τ̄*^−1^;^26^ hence the effective Péclet number grows at Pe ∼ *τ̄*. We increase, therefore, *τ̄* to probe this regime. We remark, incidentally, that it is also possible to modulate the effective diffusivity by changing the mean tumbling angle; thus in real systems one must expect that the statistics of reorientations also affects the polar order. Indeed, for *τ̄* ≈ 30, we observe from the joint PDF, shown in Fig. 6(A) as a colour map, a larger probability of finding downward oriented particles close to the wall, whereas the opposite trend appears at higher elevations, which means that in the bulk the active particles preferentially swim upwards (*i.e.* against gravity), in line with the theoretical results.^9^ To highlight this behaviour we also report the orientation PDFs, *P*(z*, ê_*z*_)/*ρ*(z*), as histograms at two heights, z* = 2*R* and z* = 30*R*.

It is worth noting that the chosen value of *τ̄* is comparable, for instance, with that of *E. coli*, for which, having *v*_*p*_ ≈ 25 μm s^−1^ and a typical run time of ∼1 s ^25,37^ and size *R* ∼ 1 μm, one gets *τ̄* = *v*_*p*_*τ*/*R* ≈ 25.

For *β* ≠ 0 we expect this scenario to break down, because the generation of fluid motion acts as an effective source of *noise*; in fact, we observe that, close to the wall, the PDF is peaked around ê_*z*_ ≈ 0 for *β* < 0, and it is bimodal (with a higher peak at ê_*z*_ ≈ −1) for *β >* 0, while in the bulk it is rather uniform in both cases (panels (B) and (C) of Fig. 6). As anticipated in the previous section, such different orientational ordering between pushers and pullers turns out to have an impact also on the swimmers’ distribution in space, as indicated by the sedimentation profiles.

## 6 Conclusions

We have presented a computational study of suspensions of run-and-tumble squirmers under gravity. Thanks to the built-in properties of the mesoscopic approach adopted we could take into account both the finite size of particles and the hydrodynamics of the solvent. In the case of potential swimmers, agreement has been found with theoretical predictions regarding (i) the dependence of the density profiles on the activity/gravity ratio and (ii) the emergence of a polar order from the inspection of distributions of particle orientations. We have provided evidence, not reported so far, that, for pushers and pullers with large enough *β*, the hydrodynamic flows induced by their collective motion determine sedimentation profiles that cannot be understood in terms of a single swimmer response to the gravitational field. This feature appeared particularly distinctive in the emblematic case of shakers, whose sedimentation problem was never studied before. We have, then, proposed a generalised diffusive model, based on the concept of a height dependent *collective* effective temperature that proved to be able to recover the observed sedimentation profiles. Moreover, we showed that the profiles of pullers and shakers with positive *β* may develop a tail in the bulk of roughly constant density, signalling the presence of a supernatant, depending on the activity/gravity ratio (the parameter *χ*_2_) but also on the system geometry (the aspect-ratio *Γ*). The emergence of such a supernatant, no previous observation of which we are aware of, is probably connected to the complex correlation between spatial organization of the microswimmers in the sediment and generated flows in the solvent, which deserves further investigation.

Overall, our findings, along the lines of recent studies,^10,38^ emphasize the importance of exploring, theoretically, numerically and experimentally, the full three-dimensional dynamics for the sake of a better understanding of the sedimentation phenomenology in active suspensions.

## Conflicts of interest

There are no conflicts to declare.

## Supplementary Material
